# Myeloidderived suppressor cells: Escorts at the maternal–fetal interface

**DOI:** 10.3389/fimmu.2023.1080391

**Published:** 2023-02-02

**Authors:** Bo Pang, Cong Hu, Huimin Li, Xinyu Nie, Keqi Wang, Chen Zhou, Huanfa Yi

**Affiliations:** ^1^ Central Laboratory, First Hospital of Jilin University, Changchun, Jilin, China; ^2^ Cardiology Department, First Hospital of Jilin University, Changchun, Jilin, China; ^3^ Reproductive Medicine Center, Prenatal Diagnosis Center, First Hospital of Jilin University, Changchun, Jilin, China; ^4^ Department of Clinical Laboratory, The Second Hospital of Jilin University, Changchun, China; ^5^ General Department, First Hospital of Jilin University, Changchun, Jilin, China

**Keywords:** myeloid-derived suppressor cells (MDSCs), maternal–fetal immune tolerance, embryo implantation, pregnancy complications, newborn

## Abstract

Myeloid-derived suppressor cells (MDSCs) are a novel heterogenous group of immunosuppressive cells derived from myeloid progenitors. Their role is well known in tumors and autoimmune diseases. In recent years, the role and function of MDSCs during reproduction have attracted increasing attention. Improving the understanding of their strong association with recurrent implantation failure, pathological pregnancy, and neonatal health has become a focus area in research. In this review, we focus on the interaction between MDSCs and other cell types (immune and non-immune cells) from embryo implantation to postpartum. Furthermore, we discuss the molecular mechanisms that could facilitate the therapeutic targeting of MDSCs. Therefore, this review intends to encourage further research in the field of maternal–fetal interface immunity in order to identify probable pathways driving the accumulation of MDSCs and to effectively target their ability to promote embryo implantation, reduce pathological pregnancy, and increase neonatal health.

## 1 Introduction

Recently, a population of immature cells derived from myeloid progenitors, i.e., myeloid-derived suppressor cells (MDSCs), has entered the research spotlight (1). The molecular phenotypes of MDSCs are CD11b and Gr1 in mice, whereas it is HLA-DR^low/−^CD33^+^ in humans. These can be subdivided into the monocytic and granulocytic subtypes (M-MDSCs and G-MDSCs, respectively). Traditionally, they are defined as CD11b^+^Ly6C^high^Ly6G^−^ and CD11b^+^Ly6G^+^Ly6C^low^ in mice and CD11b^+^CD33^+^CD14^+^HLA-DR^−/low^ and CD11b^+^CD33^+^CD15^+^/CD66^+^HLA-DR^−^ in humans, respectively (2). Recently, a number of novel markers and subsites of MDSCs have emerged, as shown in **Table 1**. MDSCs exhibit significant heterogeneity because M-MDSCs and G-MDSCs can further differentiate into myeloid cells. Furthermore, it is difficult to distinguish them from mature monocytes and granulocytes based on immune molecular markers (30). In addition, a great deal of attention has been focused on the immunosuppressive function of MDSCs in tumors, autoimmune diseases, organ transplantation, and infectious diseases (31–34). In tumors, MDSCs inhibit the antitumor functions of T cells and natural killer (NK) cells and promote tumor progression by accelerating angiogenesis and cell invasion (2, 35, 36). Moreover, the proportion of MDSCs is closely associated with the clinical outcomes and therapeutic effects in patients with cancer (37). Although MDSCs are short-lived cells, they play crucial roles in many diseases.

**Table 1:** Phenotypes of myeloid-derived suppressor cells (MDSCs) in mice and humans.

	G-MDSCs		M-MDSCs		Other types of MDSCs		Reference
	Mice	Human	Mice	Human	Mice	Human	
Traditional markers	CD11b^+^Ly6G^+^Ly6C^low^		CD11b^+^Ly6G^−^Ly6C^high^				(2–4)
		CD11b^+^CD33^+^CD14^−^CD15^+^CD66^+^HLA-DR^low/−^		CD11b^+^CD33^+^CD14^+^CD15^−^CD66^−^HLA-DR^low/−^			(2, 4–6)
Novel markers	Side scatter (SSC)						(7, 8)
	CD244						(9)
	CD84						(10)
	CD36						(11)
	FATP2						(12)
		CD13					(13, 14)
		CD16					(15)
		LOX1					(16)
			CD49d (VLA4)				(17)
			CD115				(18)
			CCR2				(19)
				CD11c			(20, 21)
				IL-4Rα (CD124)			(22)
				CD68^+^CD163^+^			(23)
					**Early-stage MDSCs (e-MDSCs)** CD11b^+^Gr-1^−^F4/80^−^MHCII^−^		(24)
						**e-MDSCs** Lin^−^CD3^−^CD14^−^CD15^−^CD19^−^CD56^−^CD33^+^HLA-DR^−^	(25)
						**MDSC-like fibrocytes** Collagen type-1^+^CD15^+^CD45^dim^CD34^−^CD14^−^/CD11c^hi^CD123^−^CD14^−^	(26, 27)
					**Fibrocytic-MDSCs (f-MDSCs)** CD11b^+^Gr-1^+^FAP^+^		(28)
						**f-MDSCs** CD33^+^IL-4Rα^+^	(28, 29)

G-MDSCs, granulocytic myeloid-derived suppressor cells; M-MDSCs, monocytic myeloid-derived suppressor cells.

The function of MDSCs during reproduction remains controversial. Embryo implantation, including the balance of immune cells at the maternal–fetal interface, reconstruction of the endometrial spiral arteries, and moderate invasion of trophoblasts, is a prerequisite for a successful pregnancy. Several studies have described how maternal–fetal tolerance is enhanced by accumulating MDSCs to suppress T-cell responses (38–40). Furthermore, the proportion of MDSCs was decreased in spontaneously aborting mice compared with controls, whereas their depletion resulted in the increased cytotoxicity of decidual NK cells (41). Even in infertile couples, the ratio of MDSCs in the peripheral blood of women who undergo *in vitro* fertilization (IVF) is considered an essential factor in predicting the outcomes of pregnancy (42, 43). These findings might provide new insights into immune-related miscarriage and IVF failure. In addition, several studies have further demonstrated the accumulation and augmentation of MDSCs in newborn humans and mice (44–46). There is growing acceptance that MDSCs manipulate the maternal–fetal immune microenvironment to promote and sustain embryo implantation, protect the fetus during gestation, and keep the newborn healthy.

In this review, we discuss the evidence concerning the association of MDSCs with embryo implantation, pregnancy complications, and newborn health and explore the potential relationship between MDSCs and the maternal–fetal immune microenvironment.

## 2 MDSCs in embryo implantation

The first steps to a successful pregnancy are embryo localization, adhesion, and invasion of the endometrium. As a type of semi-allogeneic transplantation, the fetal trophoblasts in the maternal–fetal interface interact with the maternal decidual stromal cells, decidual glandular epithelial cells, and numerous immune cells (47, 48). The maternal–fetal immune microenvironment plays a decisive role during these events. A balance between the pro- and anti-inflammatory cells (T cells, NK cells, and MDSCs) is crucial for successful implantation and placentation (40, 49–53). In addition to protecting the fetus from maternal immune attacks, immune cells play critical roles in the migration and invasion of trophoblasts and in forming decidual blood vessels (54). The maternal corpus luteum and fetal placenta can signal the production of high levels of estrogen and progesterone (55), and interactions involving hormones and immune cells cannot be ignored.

### 2.1 Immunosuppressive function of MDSCs

Immunotolerance is vital in the maternal immune system in order to accept the embryo. As a population of novel immunoregulatory cells, MDSCs have received increasing attention during early pregnancy rather than in cancer and autoimmune diseases. Besides investigations into the frequency and induction of MDSCs in pregnant human and mouse models, studies have also focused on understanding their immunosuppressive effects during pregnancy. G-MDSCs and M-MDSCs exert immunosuppressive functions *via* different molecular mechanisms (56). M-MDSCs function primarily through the secretion of nitric oxide and arginase-1 (Arg-1) (57), whereas G-MDSCs produce reactive oxygen species (ROS) and hydrogen peroxide (58). Cytokines, growth factors, and microorganisms enhance the immunosuppressive functions of MDSCs in tumors or the inflammatory microenvironment through mediators and subsequently activate nuclear factor kappa B, signal transducer and activator of transcriptions (STATs), and other signaling pathways (59–61).

An earlier study reported that G-MDSCs comprise the dominant MDSC subset in the peripheral blood of pregnant women, producing immunosuppressive enzymes such as Arg-1 and inducible nitric oxide synthase (iNOS) (62). In an MDSC depletion pregnancy mouse model, T cells showed higher proliferation capacity without Arg-1 and ROS (63). In our preliminary research, the T cells in the peripheral blood of infertile patients exhibited higher immunosuppressive function than those in the MDSC-depleted group (43). Furthermore, it has been suggested that MDSCs reduce the T-cell responses in a cell–cell contact manner (40). In humans, a subpopulation of decidual MDSCs was first recognized by Bartmann et al. in 2015. These cells secrete high levels of immunosuppressive products, including Arg-1, iNOS, and indoleamine-2,3-dioxygenase (IDO), and produce anti-inflammatory cytokines, such as interleukin-10 (IL-10) and transforming growth factor beta (TGF-β), with the ability to inhibit T-cell proliferation (64). Although the relationship between endometriosis and embryo implantation has received increasing attention, the underlying molecular mechanisms remain unclear. Elevated G-MDSC counts positively regulate the immunosuppressive activity (increased Arg-1 and ROS levels and suppressed T-cell proliferation) and the increased number of endometrial lesions. However, MDSCs depleted with the anti-Gr-1 antibody in mice exhibited dramatically fewer lesions (65). This indicates that MDSCs could possibly reduce endometrial receptivity so as to affect embryo implantation through immune attack.

As a form of semi-allogeneic transplantation, the embryo is considered as a foreign antigen by the maternal immune system; therefore, appropriate immunosuppressive products are essential in inducing immune tolerance.

### 2.2 MDSCs shift T-cell differentiation

The maternal immune system is a complex immune paradox. The mother is sensitized by foreign embryos in early pregnancy under estrogen and progesterone stimulation. Subsequently, immune tolerance is induced to protect the fetus from maternal immune attack. In recent years, the research on maternal–fetal immunity has focused on the balance between T helper (Th) 1/Th2 cells and Th17/regulatory T cells (Tregs) (66–68). The hyperfunction of pro-inflammatory T cells, such as Th1 and Th17, has been confirmed to be associated with recurrent implantation failure (69, 70).

It is well documented that MDSCs in the decidua isolated from pregnant women can induce the expression of forkhead box P3 (Foxp3) in CD4^+^ T cells (Tregs) through the TGF-β/β-catenin signaling pathway. Furthermore, CD4^+^Foxp3^+^ T cells are associated with increased MDSC number and recruitment, resulting in a positive feedback loop (71). In addition, MDSCs have been reported to induce a shift toward the anti-inflammatory subtype of Th2 cells *via* cell–cell interactions (72). Exosomes released by G-MDSCs in pregnant women have the ability to suppress T-cell proliferation, polarize Th cells to Th2 and Tregs, and inhibit lymphocyte cytotoxicity (73). Although the exact reason remains unclear, the reduction of L-selectin expression in naive T cells has been considered to inhibit their trafficking toward lymph nodes (38). Reducing the frequency of MDSCs leads to T-cell responses that enhance and elevate the ratio of Th1 to total T cells in pregnant women (74). In addition to T cells, multiple types of effector immune cells, including macrophages, uterine natural killer (uNK) cells, and immature dendritic cells (iDCs), play substantial roles in the induction of maternal–fetal immunotolerance (39, 75, 76).

### 2.3 MDSCs affect NK cell cytotoxicity

NK cells represent one of the dominant innate lymphoid cell types that exert cytotoxic effects and primarily contribute to the killing of pathogen-infected cells and tumor cells (77). These cells can primarily be categorized into two subtypes: peripheral blood NK (pbNK) cells and tissue-resident NK (trNK) cells. pbNK cells are identified as CD3^−^CD56^+^ in humans, whereas trNK cells exhibit various phenotypes and signatures (78, 79). Therefore, the CD45^+^CD56^+^Lin^−^ cells in humans or the CD45^+^Lin^−^NK1.1^+^NKp46^+^ cells in mice are defined as uNK cells. Owing to the unique structure of the uterus, uNK cells are mostly distributed in the endometrium. uNK cells are also known as decidual NK cells (80). In contrast to the cytotoxicity in pbNK cells, uNK cells facilitate decidualization and placenta formation by remodeling the extracellular matrix and endometrial stroma vessels (81).

NK cells can trigger targeted cell death by releasing cytotoxic granules, such as granzymes and perforin. Furthermore, NK cells are also employed to recognize and eliminate target cells through the NK group protein 2D-activating NK receptor (NKG2C). A study on mouse models identified that MDSC depletion results in the upregulation of the embryo resorption rates and a decrease in the uNK cell counts. Furthermore, MDSC depletion increased the cytotoxicity of uNK cells by upregulating perforin and granzyme B in the cytoplasm and NKG2C on the cell surface (38, 41, 63). It cannot be ignored that MDSCs also represent something of a double-edged sword during pregnancy because of the immunosuppressive function of pbNK cells. In a pregnant mouse model with breast cancer, MDSC accumulation led to the inhibition of NK cell activity and the subsequent promotion of tumor metastasis and disease progression (82).

### 2.4 MDSCs promote angiogenesis

Epidemiological and experimental research confirmed that an imbalance in circulating angiogenic factors directly leads to embryo implantation failure and maternal pregnancy complications, such as preeclampsia (83, 84). Although several potential cellular immunities associated with MDSCs at the maternal–fetal interface have been identified, MDSCs are related to vascular remodeling during embryo implantation and placentation. For instance, it is well documented that MDSCs support angiogenesis in tumor environments (36, 85, 86). Adequate invasion of extravillous trophoblasts into the endometrium with dilated and reconstructed spiral arteries in the myometrium so as to maintain an adequate blood supply is a prerequisite for a successful pregnancy. It has been shown that MDSCs play crucial roles during placental formation, similar to that during tumorigenesis (87). However, there is no direct evidence suggesting that MDSCs promote angiogenesis during human placentation. In rats, the MDSC-derived vascular endothelial growth factor (VEGF) has been confirmed to promote the development of maternal uterine spiral arteries and placenta to elevate the reception of implantation (88). In patients undergoing IVF, elevated serum VEGF levels were positively related to the MDSC ratio. Furthermore, the VEGF level and MDSC ratio positively correlated with the pregnancy rates (43). In addition to VEGF, MDSCs have also been discovered to secrete various bioactive factors, including CXC chemokine receptor 2 (CXCR2), CXCR4, IL-6, TGF-β, and metalloproteinases, that could facilitate tumor migration and metastasis and promote angiogenesis (36). G-MDSCs in the decidua with a high expression of CXCR2 have also been found at the maternal–fetal interface and supposedly induced angiogenesis (89, 90). In patients with spontaneous miscarriage, VEGF activated the VEGF or MEK/ERK signaling pathways in the villi to govern uterine angiogenesis and vascular remodeling during pregnancy (91, 92). In general, promoting angiogenesis may be another role of MDSCs in promoting embryo implantation.

### 2.5 Mutual promotion of MDSCs and trophoblasts

The embryo must experience the cleavage stage and the blastocyst stage and finally implant into the endometrium. MDSCs are more abundant in the peripheral blood and decidua of pregnant women and mice than in those of non-pregnant individuals (74, 81). In one of our preliminary experiments to investigate the relationship between MDSCs and trophoblasts, we showed that the proportion of MDSCs in the peripheral blood of IVF patients was positively correlated with pregnancy outcomes (43, 62). These findings suggest that MDSCs enhance trophoblast cell activity. Trophoblasts are also effective players in the expansion of MDSCs. An *in vitro* study using a trophoblast cell line (HTR8/SVneo) reported the successful triggering of peripheral CD14^+^ myelomonocytic cell differentiation into MDSCs with high expression of IDO1 and Arg-1. Notably, these cells also expressed higher levels of STAT3, indicating the potential mechanisms underlying trophoblast-induced MDSC differentiation (93). Together, these findings reveal the positive feedback loop of trophoblast implantation and MDSC recruitment at the maternal–fetal interface.

### 2.6 Expansion and differentiation of MDSCs under conditions of hormone stimulation

During gestation, the maternal corpus luteum and fetal placenta can relay to produce high levels of estrogen and progesterone (55), and the interactions between hormones and MDSCs cannot be ignored. The MDSC count and frequency steadily increase during pregnancy and peak in the second trimester, showing parallel changes with the circulating estradiol and progesterone levels. Exosomes isolated from the peripheral blood MDSCs of pregnant women exhibited various abilities involving differentiation and suppressive functions according to the stage of gestation (73). Growing evidence has shown that the recruitment and the accumulation of MDSCs depend on estradiol signaling in cancer patients (94). During human gestation, high estradiol and progesterone levels considerably facilitate the ratio and suppressive functions in both G-MDSCs and M-MDSCs *via* the STAT3 signaling pathway (40, 95). Estradiol alone may induce an increased VEGF expression in MDSCs to promote maternal uterine spiral arteries and placental development in rats (88, 96). Similar to the above-mentioned hormones, human leukocyte antigen G (HLA-G), a molecule secreted and expressed by several cells in the maternal–fetal interface, is also a crucial player in MDSC accumulation in the decidua (97). It usually binds to immunoglobulin-like transcript 4 and induces the expansion and differentiation of MDSCs in peripheral blood mononuclear cells through STAT3 signaling (98, 99). A recent study has shown that the estradiol receptor negatively modulated the expression of hypoxia-inducible factor-1 alpha (HIF-1α) (100). HIF-1α deficiency at the maternal–fetal interface leads to a decrease in the MDSC counts. Furthermore, silencing the expression of HIF-1α in MDSCs results in an impaired immunosuppressive function and renders them susceptible to apoptosis (101). Therefore, hormones protect and ensure embryo implantation and regulate the function of MDSCs through multiple pathways.

### 2.7 Lipid metabolism of MDSCs

Cellular energy metabolism is currently a critical area of focus in scientific research. Understanding the role of energy metabolism could help in better comprehending the fate of MDSCs during gestation or in pathological processes. An association between MDSCs and lipid metabolism was first recognized in 2011 by Xia et al. (102). The authors identified that MDSCs accumulated in the fat tissue, the liver, and peripheral blood, with pro-inflammation and reestablishment of metabolic homeostatic functions, even increasing insulin resistance. In contrast, the adoptive transfer of MDSCs reduced obesity-associated inflammation and improved insulin sensitivity (103). In humans, the M-MDSC counts were higher in the peripheral blood of obese men compared to those in the control group (104). Mechanistically, excessive adipocytes secreted pro-inflammatory mediators, including prostaglandin E2, tumor necrosis factor alpha (TNF-α), interleukin 6 (IL-6), and IL-1β (105), and MDSCs were recruited to the adipose tissue, subsequently resulting in a substantially worse immune response cascade (106–108). Dyslipidemia may also lead to the accumulation and immunosuppressive capacity enhancement of MDSCs, indicating that exogenous lipids may be taken up by MDSCs (11). In patients experiencing unexplained recurrent pregnancy loss, G-MDSC differentiation was associated with the pSTAT3/FABP5/PGE2 pathway (109). These findings are instrumental in understanding the physiological mechanisms of immune tolerance in pregnancy.

## 3 MDSCs during pregnancy

Recently, the numbers of studies investigating the role of MDSCs during pregnancy have increased. MDSCs are accumulated in a time-dependent manner during gestation (62, 74). Abnormal pregnancies and pregnancy complications, including spontaneous miscarriage, intrauterine growth restriction (IUGR), and preeclampsia (PE), are also closely related to an imbalance in the maternal–fetal immunological tolerance (51, 110, 111). As reviewed and discussed previously, MDSC deficiency may participate in such adverse events.

### 3.1 MDSC deficiency leads to spontaneous miscarriage

Spontaneous miscarriage is the loss of a pregnancy before viability with no manual intervention. This condition occurs in up to 20% of recognized pregnancies (109). As listed above, there is a reciprocal causation between MDSC deficiency and spontaneous miscarriage in the first or the second trimester. A reduction in the MDSC ratio, accompanied by a decrease in the levels of Arg-1, iNOS, IL-10, and TGF-β, has been observed in a spontaneous abortion mouse model compared to controls (41). The proportion of MDSCs was significantly increased in the decidua of early pregnant women compared to non-pregnant women (71, 112). Decidual G-MDSC apoptosis is mediated by TNF-related apoptosis-induced ligand (TRAIL) in a caspase 3-dependent manner. Notably, downregulated expression of the TRAIL receptor DcR2 leads to increased G-MDSC apoptosis, and this is responsible for spontaneous miscarriage (113). Generally, high levels of estradiol and progesterone lead to the recruitment of MDSCs, and an increase in their counts, during gestation. MDSCs manipulate the outcomes of pregnancy by producing immunosuppressive molecules (62, 74), regulating immune tolerance (96, 114), promoting angiogenesis (95), and inducing trophoblast implantation (40, 63).

### 3.2 MDSC deficiency results in pregnancy complications

IUGR represents an abnormal pregnancy and is the second leading cause of perinatal morbidity and mortality, affecting 5% of pregnancies (114). IUGR is one of the major causes of spontaneous miscarriage, prematurity, stillbirth, and even infant mortality (115). On the other hand, PE is characterized by new-onset hypertension, usually occurring after 20 weeks of gestation and affecting 3%–8% of pregnancies (116). The causes of IUGR and PE are multifactorial, including maternal age and nutrition, thrombophilia, placental dysfunction, and fetal factors (117). Placental dysfunction includes uteroplacental insufficiency, vessel thrombosis, placenta or spiral artery arteritis, chronic villitis, and umbilical cord abnormalities (118, 119). There is still a lack of IUGR and PE animal models; therefore, investigations have been primarily conducted in humans. As mentioned previously, G-MDSCs are observed in all stages of pregnancy and are accompanied by high cellular levels of Arg-1 and ROS activity; however, patients with IUGR exhibit a significantly lower suppressive activity. Moreover, the frequency of G-MDSCs is negatively correlated with adverse outcomes in newborns from pregnancies with IUGR (120). The MDSC ratio and serum Arg-1 levels in patients with PE are much lower than those in healthy pregnant women (111). Furthermore, the frequency of T-cell immunoglobulin and mucin domain-containing protein 3 (TIM3)^+^ MDSCs is also more elevated in patients with PE than in healthy controls, and the Tim-3/galectin-9 pathway is considered to modulate the function of MDSCs so as to affect the pathogenesis of PE (121). We speculate that adverse pregnancy outcomes associated with IUGR and PE may be related to immune imbalance, angiogenesis disorders, and shallow placental implantation caused by MDSC deficiency during early pregnancy.

## 4 MDSCs in postpartum and newborns

MDSCs play an immunosuppressive role in tumors (122, 123) and a pro-inflammatory role in autoimmune diseases (124, 125). Similarly, there is a shift from the early anti-inflammatory profiles that contribute to embryo implantation to a late pro-inflammatory cytokine profile that promotes fetal delivery (126). Notably, the MDSC counts decline rapidly in the peripheral blood of women after parturition (62). A transcriptome study that compared pregnancy-induced MDSCs to tumor-induced MDSCs revealed similar transcriptomes in both conditions. However, the antimicrobial-associated protein levels were higher in pregnancy-induced MDSCs (127). Furthermore, the MDSCs in neonates exhibited antimicrobial activity by secreting higher levels of cathepsin G, neutrophil cytosolic factor 1, S100 calcium-binding protein A8/A9, myeloperoxidase, lysozyme, neutrophil elastase, and lipocalin 2 (127). Similarly, the MDSCs in breast milk have also been found to protect infants from necrotizing enterocolitis and nosocomial bacterial infections (128). It appears that the MDSCs transferred from the mother to the fetus during delivery *via* the umbilical cord and breast milk harbor antimicrobial effects in infants.

In addition to this protective function, MDSCs may also play a negative role in postnatal immune development, increasing susceptibility to infections in neonates. Estradiol levels are high in newborns, and there is estradiol-dominant MDSC recruitment and augmentation. Among infants, preterm babies show elevated estradiol levels in serum compared to full-term babies, while the MDSCs in umbilical cord blood also exhibit an abnormal increase (129). Premature infants are more susceptible to necrotizing enterocolitis, bronchopulmonary dysplasia, and sepsis (44, 127, 128). The underlying mechanisms may be associated with immune regulation activity and the immunosuppressive characteristics of MDSCs.

During the neonatal period, a balance between immune attack and tolerance is extremely important (130). Generally, the immunosuppressive functions of umbilical cord blood MDSCs are to inhibit the differentiation and proliferation of Th1 and Th17 cells by producing Arg-1 and ROS and inducing cell–cell contact and to enhance the accumulation of immune regulatory Th2 cells and Tregs (131–134). Furthermore, MDSCs also suppress the cytotoxic NK cells through cell–cell interactions (131). When an anti-inflammatory immune reaction is activated (represented by Th2 and Treg hyperfunction), the newborn will not exhibit excessive immune attack when exposed to various antigens, bacteria, and viruses. However, in preterm infants, the high frequency of MDSCs enhances immune tolerance, leading to insufficient immune responses and neonatal diseases (46, 134–136).

## 5 Conclusion

**Figure 1:**
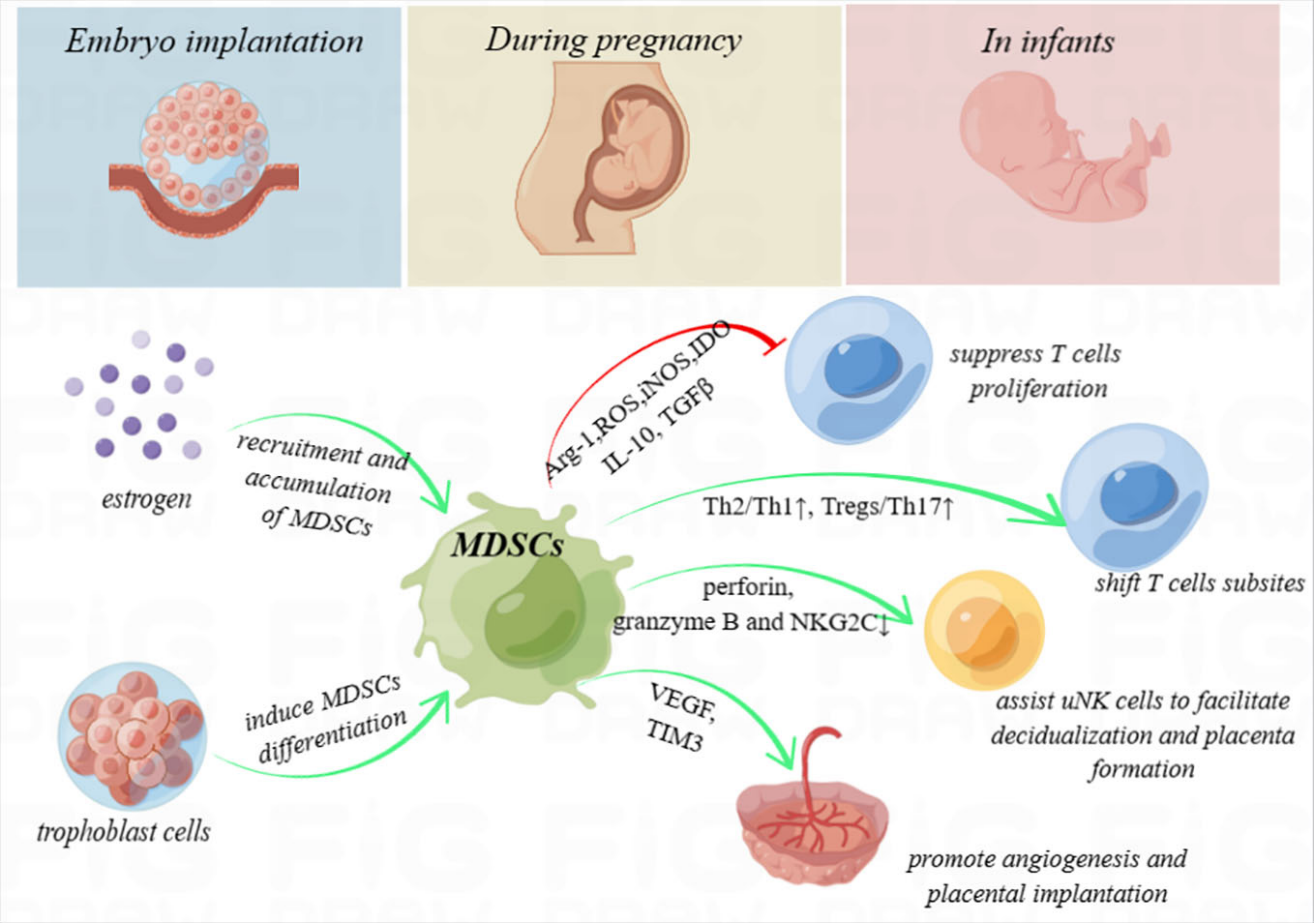
Schematic diagram. Myeloid-derived suppressor cells (MDSCs) secrete high levels of immunosuppressive products, including arginase-1 (Arg-1), reactive oxygen species (ROS), inducible nitric oxide synthase (iNOS), and indoleamine-2,3-dioxygenase (IDO), and produce anti-inflammatory cytokines, such as interleukin 10 (IL-10) and transforming growth factor beta (TGF-β), with the ability to inhibit T-cell proliferation. In addition, MDSCs shift the T helper (Th) 1/Th2 cell and Th17/regulatory T cell (Treg) subsets to exert immunoregulation function. They also enhance uterine natural killer (uNK) cells to facilitate decidualization and placenta formation by downregulating perforin and granzyme B in the cytoplasm and NK group protein 2 D‐activating NK receptor (NKG2C) on the cell surface and promote angiogenesis and placental implantation through the VEGF and TIM3 pathway. During pregnancy, high levels of estrogen and trophoblasts may recruit, accumulate, or even induce the differentiation of MDSCs. MDSCs deficiency is considered to be associated with embryo implantation failure, spontaneous miscarriage, intrauterine growth restriction, and preeclampsia. Furthermore, MDSCs even play crucial roles to enhance immune tolerance in neonates.

The immune environment at the maternal–fetal interface is complex and mysterious. MDSCs, as immunoregulatory cells with both pro-inflammatory and anti-inflammatory functions, are well documented to act as an escort throughout pregnancy (schematic diagram shown in **Figure 1**). Identifying the molecular mechanisms underlying the angiogenesis promotion and trophoblast implantation-inducing functions of these cells will enable precision therapy. During the window of implantation, intra-uterus transfusion of induced MDSCs or augment MDSCs in the decidua using multiple cytokines may provide tangible clinical benefits, such as elevate the live birth rate in recurrent embryo implantation failure, decrease pregnancy complications, and promote neonatal health. However, whether immunosuppression during pregnancy promotes the occurrence and progression of tumors remains uncertain. Further studies focusing on this aspect still need to be conducted.

## Author contributions

BP, CH, HL, XN, and KW wrote the manuscript. CZ and HY revised the manuscript. All authors contributed to the article and approved the submitted version.
